# Expert Tool Users Show Increased Differentiation between Visual Representations of Hands and Tools

**DOI:** 10.1523/JNEUROSCI.2489-20.2020

**Published:** 2021-03-31

**Authors:** Hunter R. Schone, Roni O. Maimon-Mor, Chris I. Baker, Tamar R. Makin

**Affiliations:** ^1^Institute of Cognitive Neuroscience, University College London, London, United Kingdom WC1N 3AZ; ^2^Laboratory of Brain and Cognition, National Institute of Mental Health, National Institutes of Health, Bethesda, Maryland 20892; ^3^WIN Centre, Nuffield Department of Clinical Neuroscience, University of Oxford, Oxford, United Kingdom OX3 9DU; ^4^Wellcome Trust Centre for Neuroimaging, University College London, London, United Kingdom WC1N 3AR

**Keywords:** embodiment, experts, fMRI, neuroimaging, plasticity, tools

## Abstract

The idea that when we use a tool we incorporate it into the neural representation of our body (embodiment) has been a major inspiration for philosophy, science, and engineering. While theoretically appealing, there is little direct evidence for tool embodiment at the neural level. Using functional magnetic resonance imaging (fMRI) in male and female human subjects, we investigated whether expert tool users (London litter pickers: *n* = 7) represent their expert tool more like a hand (neural embodiment) or less like a hand (neural differentiation), as compared with a group of tool novices (*n* = 12). During fMRI scans, participants viewed first-person videos depicting grasps performed by either a hand, litter picker, or a non-expert grasping tool. Using representational similarity analysis (RSA), differences in the representational structure of hands and tools were measured within occipitotemporal cortex (OTC). Contrary to the neural embodiment theory, we find that the experts group represent their own tool less like a hand (not more) relative to novices. Using a case-study approach, we further replicated this effect, independently, in five of the seven individual expert litter pickers, as compared with the novices. An exploratory analysis in left parietal cortex, a region implicated in visuomotor representations of hands and tools, also indicated that experts do not visually represent their tool more similar to hands, compared with novices. Together, our findings suggest that extensive tool use leads to an increased neural differentiation between visual representations of hands and tools. This evidence provides an important alternative framework to the prominent tool embodiment theory.

**SIGNIFICANCE STATEMENT** It is commonly thought that tool use leads to the assimilation of the tool into the neural representation of the body, a process referred to as embodiment. Here, we demonstrate that expert tool users (London litter pickers) neurally represent their own tool less like a hand (not more), compared with novices. Our findings advance our current understanding for how experience shapes functional organization in high-order visual cortex. Further, this evidence provides an alternative framework to the prominent tool embodiment theory, suggesting instead that experience with tools leads to more distinct, separable hand and tool representations.

## Introduction

Experience using tools is commonly thought to lead to an integration between the neural representations of the body and the tool, a process known as embodiment (Maravita and Iriki, 2004). While theoretically appealing, there is little direct evidence for tool embodiment at the neural level. Previous research assessing tool embodiment, such as the influential work of Iriki and colleagues (Iriki et al., 1996), measured whether tool use affects the visual representation of hand-centered space (e.g., multisensory peripersonal space; Maravita and Iriki, 2004). However, this is an indirect measure of hand representation and is therefore open to alternative interpretations (Holmes, 2012). Additionally, a more recent tool embodiment approach using electroencephalography (EEG) examined how tactile information carried by a hand-held tool is processed by the somatosensory system as compared with the hand itself (Miller et al., 2019). But considering that the tool is held by the hand, it is not clear whether this low-level representation is actually attributable to the tool, or more likely, to the mechanoreceptors in the hand that mediate this information. As such, there is still not a strong proof of concept in the literature that tool use leads to sensory embodiment.

Here, we used functional magnetic resonance imaging (fMRI) brain decoding to directly quantify similarities between visual representations of hands and tools in expert tool users and novices. We studied individuals with extensive experience using a litter picking tool (expert tool users) as well as a group of novice litter picker users. We specifically chose to study expert tool users, based on the assumption that the extensive tool use of the experts would make them most likely to embody their tools. During fMRI scans, participants viewed first-person videos depicting grasps performed by either a hand, litter picker, or a non-expert grasping tool (tongs). Using representational similarity analysis (RSA), differences in the representational structure across hands and tools were measured within occipitotemporal cortex (OTC). We specifically focused on OTC because it contains spatially overlapping, but distinct, representations for hands and tools (Bracci et al., 2012). OTC has also been closely linked in visuomotor (Orlov et al., 2010) and multisensory hand representations (Gentile et al., 2013) and has also been associated with hand embodiment under the rubber hand illusion (Limanowski et al., 2014). As such, it provides a perfect test bed for investigating tool embodiment. Additionally, to test whether a different result would potentially be observed within neural structures directly implicated in motor planning and execution for hand use and tool use (Gallivan et al., 2013), an exploratory analysis was performed in left parietal cortex. We focused on the left hemisphere because motor planning/tool use has been shown to be left-lateralized in parietal cortex (Brandi et al., 2014; Gallivan and Culham, 2015).

**Figure 1.**
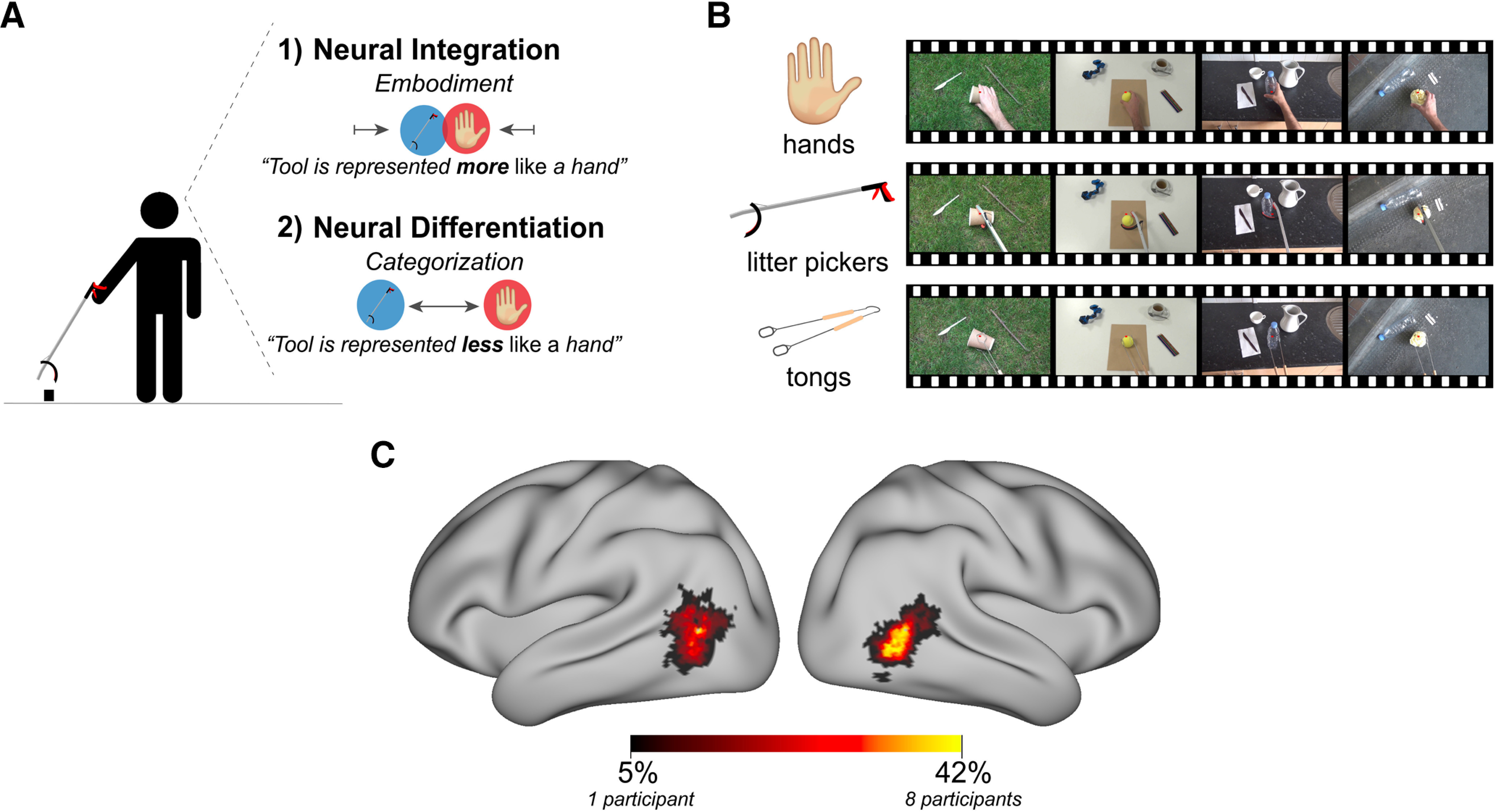
Neuroimaging hypotheses and experimental paradigm. ***A***, An illustration of the predictions generated by the proposed hypotheses for the neuroimaging experiment. Under the first, embodiment prediction, extensive tool use could lead to representations of hands and tools to become neurally integrated, such that tools are represented more similarly to hands, suggesting that tools are embodied. A second prediction is that experts will show greater categorization of representations of hands and tools, such that the neural representations for hands and tools would become differentiated and more dissimilar to each other. This would suggest that perhaps that visual experience with tools leads to an increased sharpening of the representation. ***B***, Examples of the video stimuli shown during the fMRI scan depicting grasping actions performed by each effector category: hands, litter pickers, or tongs [the videos can be downloaded on the Open Science Framework (OSF) at https://osf.io/p4q3y/]. To control for any potential laterality effects, the stimuli included both left and right-handed versions. ***C***, ROI probability map for all participants (*n* = 19) showing hand and tool selective OTC, defined using independent functional data. For each participant and hemisphere, the top 100 most activated voxels of OTC were selected based on a hands + tools > objects + low-level visual stimulus contrast. ROIs from all participants were superimposed. Warmer colors represent voxels that were included in a greater number of individual ROIs. Group-specific probability maps of OTC can be downloaded on the Open Science Framework at https://osf.io/p4q3y/.

Under the theoretical framework that defines neural embodiment as the successful integration of brain resources typically devoted to control the body to represent and operate external objects (e.g., tools, prosthetic limbs; de Vignemont, 2011; Makin et al., 2017), we proposed three predictions for what we might observe: (1) experts could represent the expert tool more like a hand compared with novices, i.e., neural embodiment, (2) experts could represent the expert tool less like a hand compared with novices, i.e., neural differentiation or (3) experts could show no differences compared with novices (Fig. 1*A*). Interestingly, we found that, contrary to the neural embodiment theory, expert tool users represent the expert tool less like a hand, i.e., greater neural dissimilarity between the visual representations of the expert tool and hands within OTC, compared with the novices. Using Crawford and Howell's (1998) method, a modified *t* test, we independently replicated this effect in five of seven individual expert litter pickers, as compared with the novices. Further, we found that this result could not be explained by the low-level representational structure captured in primary visual cortex (V1). An exploratory analysis in left parietal cortex revealed a similar pattern as OTC. These findings provide a novel framework for how tool use shapes the representational structure of hands and tools, such that extensive tool use leads to a more distinct tool representation, as compared with the hand, throughout the visuomotor network. Collectively, this evidence provides an important alternative framework to the tool embodiment theory.

## Materials and Methods

### Participants

To identify “expert” litter pickers, recruitment adverts were distributed with multiple relevant individuals/groups: sanitation supervisors stationed in London Underground stations (e.g., King's Cross St. Pancras, Westminster, Camden, Russell Square etc.), Heads of Parks and Sanitation at several UK city councils (e.g., Islington, Camden, Brighton, and Hove), and with several volunteer litter picking organizations: Keep Britain Tidy, Litter Action, CleanupUK, Helping Hand Environmental, and the Dorset Devils. From these advertisements, 52 respondents were screened via a telephone interview or online survey. From this group, 13% of respondents [*n* = 7; mean age (SD) = 47 (8.11), four females, all right-handed, mean years of education (SD) = 15.9 (1.57)] were invited to participate in the study, based on their litter picking usage being above a minimum threshold (composite score of their previous litter picking use and their current litter picking usage) and compatibility with MRI safety regulations. We also recruited a group of novices matched in age [*n* = 15; mean age (SD) = 43 (7.39), three females, 1 left-handed, mean years of education (SD) = 14.8 (1.86)]. All participant demographics are reported in Table 1. Recruitment was conducted in accordance with University College London's research ethics committee (Ref: 9937/001). Informed consent and consent to publish was obtained in accordance with ethical standards of the Declaration of Helsinki (1964). Three novices were excluded from fMRI data analysis because they did not complete all of the functional runs, because of feelings of anxiety and claustrophobia.

**Table 1.** Participant demographics

Subject	Gender	Age	Years of education	Litter picker usage	Years litter picking
EXP01	F	53	19	2.5 d/week (1.5 h/d)	1.5
EXP02	M	53	15	4.5 d/week (1.5 h/d)	10
EXP03	M	46	15	4 d/week (1.5 h/d)	0.5
EXP04	F	47	15	1.5 d/week (2 h/d)	6
EXP05	M	56	15	1 d/week (1 h/d)	3
EXP06	F	36	17	7 d/week (2.5 h/d)	2
EXP07	F	36	15	3.5 d/week (1 h/d)	4

Expert litter pickers (EXP).

### Litter picking usage measurements

Participants were asked to estimate their frequency of using a litter picking on a weekly and daily basis, as well as to estimate how long they have been using a litter picker. Litter picking usage habits are summarized below in Table 1. Participants were not asked to report their previous experience with the non-expert tool (tongs).

### Experimental design

#### fMRI task stimuli

For the main functional task, participants viewed first-person videos of grasping actions being performed using three different effector categories: hands, litter pickers (expert tool) and tongs (non-expert tool). The stimuli included 48 unique videos. Of the 48 videos, there were 16 videos for each effector category. Half of the videos (eight per effector) were presented as left-handed and the other half as right-handed. For the 8 videos for each effector category, videos varied in multiple features: scene context [common scenes typical for hand or tool actions: street (tool), grass (tool), kitchenette (hand), desk (hand)], as well as the size of the object being grasped (small vs large; for example, a small object used was a train ticket and a large object used was a tennis ball; to access all of the videos see https://osf.io/p4q3y/). A fourth effector, prosthetic hands, was also included in the design. However, this condition was included as part of a separate study involving amputee participants.

Separately, for the functional localizer scan, participants viewed videos of tools, hands, and two types of control categories: objects and low-level visual control stimuli (to access the full functional localizer video see https://osf.io/p4q3y/).

#### fMRI task design

For the main functional task, the presentation of the stimuli was counter-balanced across the four functional runs, to best control for pairwise order effects. Each functional run was 7 min 26 s in length. Within each run, each video was presented once. Each video was displayed for 3.0 s, followed by 2.5 s of a red fixation point against a gray background. Additionally, catch trials were introduced to keep subjects engaged throughout the scan, where an image of a leprechaun face would randomly appear on the center of the screen. Participants were instructed (before starting the task) to wiggle their toes whenever a leprechaun face appeared. These trials were modeled separately and excluded from further analyses. The videos were constructed using MoviePy, a python package for video editing (https://zulko.github.io/moviepy/). Stimuli were presented on a screen located at the rear end of the MRI scanner and were viewed through a mirror mounted on the head coil. The videos were presented via VLC player (https://www.videolan.org/vlc/) on a Dell Latitude laptop.

For the functional localizer scan, participants were instructed to maintain fixation on a cross in the center of the screen that was visible throughout the experiment. The localizer run began and ended with a 20-s fixation baseline, followed by five experimental blocks of five 21-s blocks (four experimental blocks and one baseline block), ending with another 20-s fixation baseline (for a total run duration of 9 min, 20 s). The order of blocks was semi-counterbalanced across the five sets. Each block of the video conditions was comprised of three videos of 7 s each, with each video depicting a different exemplar of the condition.

### MRI data acquisition

The MRI measurements were obtained using a 3-Tesla Quattro scanner (Siemens) with a 32-channel head coil. Anatomical data were acquired using a T1-weighted magnetization prepared rapid acquisition gradient echo sequence (MPRAGE) with the parameters: TR = 2.54 s, TE = 3.34 ms, FOV = 256 mm, flip angle = 7°, and voxel size = 1-mm isotropic resolution. Functional data based on the blood oxygenation level-dependent signal were acquired using a multiband gradient echo-planar T2*-weighted pulse sequence (Uğurbil et al., 2013) with the parameters: TR = 1.5 s, TE = 35 ms, flip-angle = 70°, multiband acceleration factor = 4, FOV = 212 mm, matrix size of 106 × 106, and voxel size = 2-mm isotropic resolution. Seventy-two slices, with a slice thickness of 2 mm and no slice gap, were oriented in the anterior commissure–posterior commissure, covering the whole cortex, with partial coverage of the cerebellum. Each of the four functional runs comprising the main task consisted of 298 volumes (7 min, 26 s). For the functional localizer, there was one functional run consisting of 374 volumes. For all functional scans, the first dummy volume of every run was saved and later used as a reference for co-registration.

### fMRI analysis

Functional MRI data processing was conducted using FMRIB's expert analysis tool (FEAT; version 6.0), part of FMRIB's software library (FSL; www.fmrib.ox.ac.uk/fsl) and Connectome Workbench (humanconnectome.org) software, in combination with MATLAB scripts (R2019b, v9.7; The MathWorks Inc), both developed in-house (including an FSL-compatible RSA toolbox; Nili et al., 2014) and as part of the RSA Toolbox (Wesselink and Maimon-Mor, 2018).

### fMRI preprocessing

Registration of the functional data to the high-resolution structural image was conducted using the boundary-based registration algorithm (Greve and Fischl, 2009). Registration of the high resolution structural to standard space images was conducted using FMRIB's linear image registration tool (FLIRT; Jenkinson and Smith, 2001; Jenkinson et al., 2002) and was then further refined using FNIRT nonlinear registration (Andersson et al., 2007a,b). The following prestatistical processing was applied: motion correction using MCFLIRT (Jenkinson et al., 2002), non-brain removal using BET (Smith, 2002), spatial smoothing using a Gaussian kernel of FWHM 3 mm for the functional task data and 5 mm for the functional hand-tool localizer, grand-mean intensity normalization of the entire 4D dataset by a single multiplicative factor, and high-pass temporal filtering (Gaussian-weighted least-squares straight line fitting, with σ = 50 s). Further, to minimize potential biases from individual runs, the functional data across the individual runs was aligned to a functional mid-space using FLIRT (Jenkinson and Smith, 2001; Jenkinson et al., 2002). This functional mid-space was later used to align the parameter estimates and residuals, from each run, to the same functional space for the RSA.

### Low-level task-based analysis

We applied a general linear model (GLM) as implemented in FEAT, to each functional run. For the main analysis, left and right-handed versions of the same videos were modeled together against rest (fixation). Time-series statistical analysis was conducted using FILM with local autocorrelation correction (Woolrich et al., 2001). The time series model included trial onsets convolved with a double γ HRF function; six motion parameters were added as confound regressors. Trials for each video condition were modeled separately, except left-handed and right-handed videos were modeled together. Indicator functions were added to model out single volumes identified to have excessive motion (>1 mm). A separate regressor was used for each high motion volume, no more than eight volumes were found for an individual run (2.1% of the entire run). Additionally, in the supplementary analysis exploring the effects of video laterality, videos were modeled separately for each effector category and whether they were left-handed or right-handed against rest (fixation) and averaged across the other features (context and object size). We further used this analysis to confirm our main analysis for group differences in effector category distances.

For the functional localizer scan, a single contrast for the conditions of interest were defined as hands + tools > objects + low-level visual stimulus. The activity patterns associated with this contrast were then used to define functional regions of interest (ROIs).

For each participant, parameter estimates of the different effector categories and GLM residuals of all voxels within the ROI were extracted from each run's first-level analysis. For each participant, the parameter estimates and GLM residuals from each run were then aligned to the functional mid-space using FLIRT (Jenkinson and Smith, 2001; Jenkinson et al., 2002). The subsequent RSA analysis was conducted within this functional mid-space.

### Defining ROIs

#### OTC

Using functional MRI data collected from a separate, independent group of controls (*n* = 20) that viewed the same functional hand-tool localizer (described above), a whole brain group activation map for the contrast hands and tools over moving objects and low-level visual stimulus was constructed. This group map revealed a large cluster covering OTC (*z*-threshold of 3.1). This cluster was isolated, binarized and registered to the functional space of the functional localizer scan using FLIRT. Since the focus of the study was on identifying hand and tool selective voxels within OTC, the analysis was restricted to individually defined ROIs within this large OTC map defined by the independent group of controls. Using the functional localizer data, for each participant in the present study, a hand and tool selective ROI within the large OTC map was defined by selecting the top 100 voxels in each hemisphere showing the strongest greatest preference to videos of hands and tools over moving objects and low-level visual stimulus for each participant. In total, the OTC ROI included 200 voxels: 100 in the left hemisphere and 100 in the right hemisphere. These individually defined ROIs were then transformed from the functional space of the functional localizer scan to the functional mid-space of the functional task scans (described above).

#### V1

The V1 ROI was derived from the Juelich Histologic Atlas (GM Visual Cortex V1 BA17 L and R) maximum probabilistic map (unthresholded). Each V1 hemisphere ROI was binarized and transformed from MNI space to the functional space of the functional localizer scan using FLIRT. To identify visually active voxels within each ROI, using the independent hand-tool functional data, the top 100 most activated voxels, in each hemisphere, were selected based on the contrast of all video conditions > baseline. In total, the V1 ROI included 200 voxels: 100 in the left hemisphere and 100 in the right hemisphere. These individually defined ROIs were then transformed from the functional space of the functional localizer scan to the functional mid-space of the functional task scans. ROIs from all participants were superimposed.

#### Left parietal cortex

As an exploratory analysis, the analysis performed in OTC was conducted in left parietal cortex. Using the functional MRI data collected from the separate, independent group of controls (*n* = 20) that viewed the same functional hand-tool localizer (described above for OTC), a whole brain group activation map for the contrast hands and tools over moving objects and low-level visual stimulus was constructed. This group map revealed a large cluster covering parietal cortex (*z*-threshold of 3.1). The left parietal cortex cluster was isolated, binarized and registered to the functional space of the functional localizer scan using FLIRT. Since the focus of the study was on identifying hand and tool selective relevant voxels, the analysis was restricted to individually defined ROIs within the large left parietal map. To identify hand and tool selective voxels within this map, the top 200 most activated voxels within the left parietal hand-tool conjunction map were selected, for each participant, based on a hands + tools > objects + low-level visual stimulus contrast. These individually defined ROIs were then transformed from the functional space of the functional localizer scan to the functional mid-space of the functional task scans. ROIs from all participants were superimposed.

### RSA

To assess the hand-tool representation structure within the ROI, we used a mutlitvariate approach, RSA, where pairwise representational dissimilarity distances between individual videos were calculated (Diedrichsen and Kriegeskorte, 2017). For each participant, parameter estimates of the individual videos and GLM residuals of all voxels within the ROI were extracted from each run's first-level analysis. To increase the reliability of the distance estimates, parameter estimates underwent multidimensional normalization based on the voxels' covariance matrix calculated from the GLM residuals. This was done to ensure that parameter estimates from noisier voxels will be down-weighted (Walther et al., 2016). Cross-validated (leave-one-run-out) Mahalanobis distances [also known as linear discriminant contrast (LDC); Nili et al., 2014; Walther et al., 2016] were then calculated between each pair of videos. Analysis was run on adapted version of the RSA toolbox in MATLAB (Nili et al., 2014), customized for FSL (Wesselink and Maimon-Mor, 2018).

**Figure 2.**
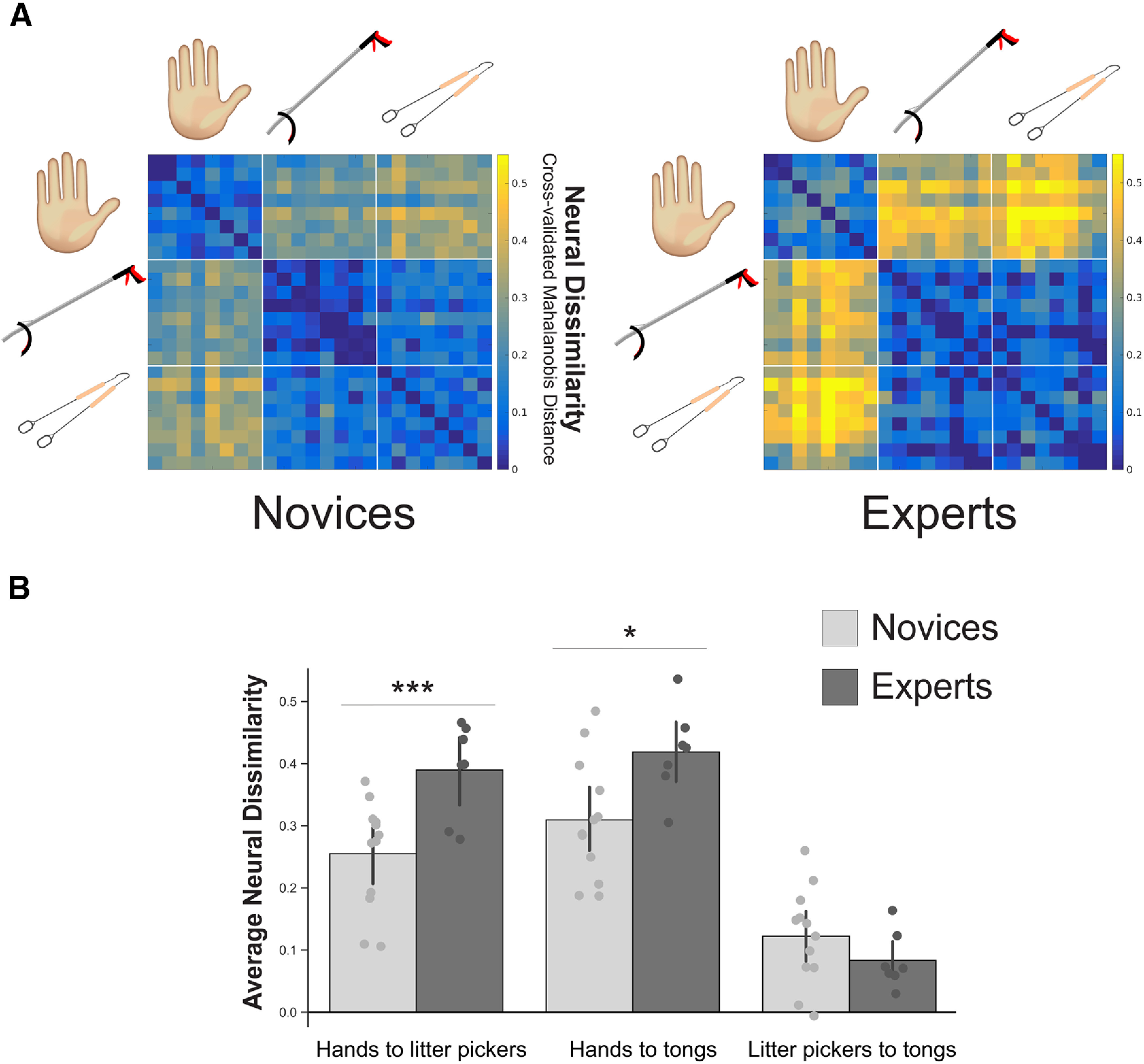
Expert tool users represent tools less like hands. ***A***, Group RDMs showing the pairwise distances (cross-validated Mahalanobis distance) between each video condition. Each element in the matrix was averaged across left and right OTC. Warmer colors indicate the conditions that evoked more dissimilar patterns of activity. Group multidimensional scaling plots derived from these group RDMs can be accessed at https://osf.io/p4q3y/. ***B***, Bar plot of individual participants for each cross-effector category distance pair: hands ↔ litter pickers, hands ↔ tongs, and litter pickers ↔ tongs. These values are generated by averaging the 8 × 8 pairwise comparison values, for each effector category pair, for each subject individually. Dark gray values reflect expert tool users (*n* = 7). Light gray values reflect novices (*n* = 12). Circles depict individual subject means. Values indicate group means ± standard error. Asterisks denote significance as follows; *p < 0.05, ***p < 0.005.

For OTC, this analysis was performed separately for each participant and ROI (left OTC, right OTC), resulting in pairwise dissimilarity distance values comparing each video condition (note that left-handed and right-handed videos were modeled together in this analysis). These distance values for each ROI were inputted into a mixed level ANOVA (described later in statistical analyses). Because of no significant interaction with ROI (left OTC, right OTC), the resulting values for left and right OTC were averaged for each participant, for visualization purposes. These distance values were then depicted as a representational dissimilarity matrix (RDM), where each element in the RDM corresponds to a single pairwise dissimilarity distance value. The group RDMs (Fig. 2*A*) were constructed through averaging each pairwise distance element in the matrix of each participant for each group (novices, experts). Additionally, multidimensional scaling plots (to access see https://osf.io/p4q3y/) were derived from these group RDMs. MDS projects the higher-dimensional RDM into a lower (2D) dimensional space. Note that MDS is presented for intuitive visualization purposes only and was not used for statistical analysis. For V1, the same analysis parameters were used, except the RSA was performed across both hemispheres. For parietal cortex, the same analysis parameters for OTC were used, except we only analyzed the left hemisphere.

For the laterality RSA analysis, the analysis was performed twice: separately for the average parameter estimates for left-handed and right-handed stimuli. Cross-validated (leave-run-out) Mahalanobis distances were calculated between the parameter estimates for each pair of conditions (e.g., for left-handed stimuli: left-handed hands, left-handed litter pickers, left-handed tongs). Specifically, for the laterality analysis performed in OTC, this was done separately in each OTC hemisphere for each participant, resulting in 4 RDMs: left-handed stimuli in left OTC, right-handed stimuli in left OTC, left-handed stimuli in right OTC, right-handed stimuli in right OTC. The group RDMs for each of these brain regions were constructed through averaging each pairwise distance element in the 3 × 3 matrix of each participant for each group (novices, experts). Again, for parietal cortex, the same analysis parameters for OTC were used, except we only analyzed the left hemisphere (i.e., no within subject-factor of ROI in the mixed-model ANOVA).

### Statistical analyses

All statistical testing was performed using IBM SPSS Statistics for Macintosh (version 24), with the exception of the Bayesian analysis which was run on JASP (version 0.11.1; Jasp Team, 2020) Tests for normality were conducted using a Shapiro–Wilk test. For statistical analyses of RSA measures in OTC, a mixed level ANOVA (after testing for normality using the Shapiro–Wilks test, *p* > 0.05) was performed with the within-subject factors: effector category distances (hands ↔ litter pickers, hand ↔ tongs, litter pickers ↔ tongs) and ROI (left OTC, right OTC) and a between subject factor group (novices, experts). For the secondary OTC analysis that controlled for low-level representational structure captured in V1, the same parameters for the OTC mixed level ANOVA described above were used; however, the average effector category distance outputted from V1 for each participant was used as a covariate. For V1, a mixed level ANOVA (after testing for normality using the Shapiro–Wilks test, *p* > 0.05) was performed with the within-subject factors: effector category distances (hands ↔ litter pickers, hand ↔ tongs, litter pickers ↔ tongs) and a between subject factor group (novices, experts). For the OTC laterality analysis, each participant's cross-effector category distances from each of the two RDMs for each ROI (left OTC, right OTC) were inputted into a mixed level ANOVA (after testing for normality using the Shapiro–Wilks test, *p* > 0.05) was performed with the within-subject factors included: effector category distances (hands ↔ litter pickers, hand ↔ tongs, litter pickers ↔ tongs), laterality (left-handed or right-handed) and ROI (left OTC, right OTC) and a between subject factor group (novices, experts).

For the left parietal cortex RSA analyses, the same ANOVA parameters were used as OTC, except there was no within-subject factor of ROI. Within all of the above analyses, to explore the group differences in pairwise effector category distance pairs, two-tailed independent samples *t* tests and two-tailed Bayesian independent samples *t* tests were performed. The Cauchy prior width was set at 0.707 (default; Keysers et al., 2020). We interpreted the test based on the well accepted criterion of Bayes factor smaller than 1/3 (Dienes, 2014) as supporting the null hypothesis. The strength of evidence was interpreted based on the classification provided in (Jeffreys, 1961), where a Bayes factor above 10 (or below 0.1) is considered as strong evidence. Additionally, as an exploratory analysis to characterize the supporting evidence for tool embodiment in left parietal cortex, one-tailed Bayesian independent samples *t* tests were performed. The alternative hypothesis was defined as “experts have smaller dissimilarity distances between hands and the expert tool (litter pickers) than novices,” i.e., novices > experts.

To test whether an individual expert litter picker's hands ↔ litter pickers distance was significantly different from the novices, we used Crawford and Howell's (1998) method which provides a point estimate of the abnormality of the individual case's distance from a control sample, as well as a confidence interval of the uncertainty associated with the point estimate (Crawford and Howell, 1998). To account for interindividual differences not directly related to hand-tool representation, we first subtracted each participant's hands ↔ litter pickers distance by their litter pickers ↔ tongs distance. The analysis was performed using the Singlims.exe program (Crawford and Garthwaite, 2002).

## Results

First, to investigate whether experience with a hand-held tool leads to tools being embodied, we recruited individuals with extensive experience using a litter picking tool (*n* = 7, identified from 52 screened litter pickers; see participant demographics in Table 1). To quantify whether the expert litter pickers neurally embody the litter picker, we used fMRI in combination with RSA to measure differences in the representational structure of hands and tools. During fMRI scans, participants viewed first-person videos of grasping actions being performed by three effector categories: hands, litter pickers (expert tool) and tongs (non-expert tool). Videos were visually matched across the effector categories. Videos also varied in multiple features: scene context [common scenes typical for hand or tool actions: street (tool), grass (tool), kitchenette (hand), desk (hand)], object sizes (small, large), and the laterality of stimuli (left-handed or right-handed; for screenshots of the videos, see Fig. 1*B*). Next, individualized hand and tool selective ROIs within OTC were independently localized for each participant by choosing the 100 OTC voxels in each hemisphere showing the strongest preference to videos of hands and tools over moving objects and low-level visual stimulus (Fig. 1*C*).

### Expert tool users show increased differentiation between hands and tools in OTC

To calculate group differences between activation patterns for hands and tools in OTC, we first computed the representational dissimilarity distances comparing each of the video conditions to every other video condition (for the RDMs for each group, see Fig. 2*A*). While participants viewed multiple video conditions for each of the three effector categories, we focused on the representational distances between effector representations, across the multiple conditions. To do this, we averaged the cross-effector category representational dissimilarity distances for each participant. This resulted in three distances per participant, one for each cross-effector category pair (hands ↔ litter pickers, hands ↔ tongs, litter pickers ↔ tongs). We entered these distances into a mixed level ANOVA: within-subject factors included the three cross-effector category distances and ROI (left OTC, right OTC), with a between-subject factor of group (experts, novices). This analysis revealed a significant two-way interaction between the effector category distances and group (*F*_(2,16)_ = 17.495, *p* < 0.001; BF_incl_ = 72.313; the three-way interaction between ROI, effector category distances and group was not significant: *F*_(2,16)_ = 1.267, *p* = 0.309; BF_incl_ = 1.088; Fig. 2*B*). This suggests that there are group differences in the representational structure (full statistical report can be accessed at https://osf.io/p4q3y/). Specifically, expert tool users represented the expert tool less like hands, i.e., experts showed increased dissimilarity distances between the expert tool (litter picker) and hands, compared with the novices (*t*_(17)_ = −3.385, *p* = 0.004, two-tailed; BF_10_ = 11). Thus, the extensive tool use of the experts leads to the visual representation of the tool to become more dissimilar to hands (not more similar). Moreover, this shift was also observed for the non-expert tool (tongs) with experts representing the tongs less like hands, i.e., experts showed increased dissimilarity distances between tongs and hands, compared with novices (*t*_(17)_ = −2.574, *p* = 0.020, two-tailed; BF_10_ = 3.1). Additionally, the two grasping tools (litter pickers ↔ tongs) were represented equally similar to each other, i.e., no significant group differences in dissimilarity distances between the litter picker and tongs (*t*_(17)_ = 1.202, *p* = 0.246, two-tailed; BF_10_ = 0.6).

Considering the small sample size of the expert litter pickers group, we next sought to test whether the observed effect in the experts could be replicated in each individual expert litter picker, as compared with the novice group. As such, one could consider each expert litter picker to be a case study and an independent replication of the effect. To test this, we used Crawford and Howell's (1998) method (a modified *t* test) to test whether each expert litter pickers' hands ↔ litter pickers distance was significantly different from the novices (Crawford and Howell, 1998). This analysis revealed that five of seven expert litter pickers showed significantly greater hands ↔ litter pickers distances (normalized by the litter pickers ↔ tongs distance), as compared with the novices (two-tailed; range of *p* values for the five experts with significant tests: 0.002 < *p* < 0.022; *p* values for the two experts with non-significant tests: 0.144 and 0.245). This analysis further confirms that expert litter pickers show increased neural differentiation between visual representations of hands and tools within OTC.

**Figure 3.**
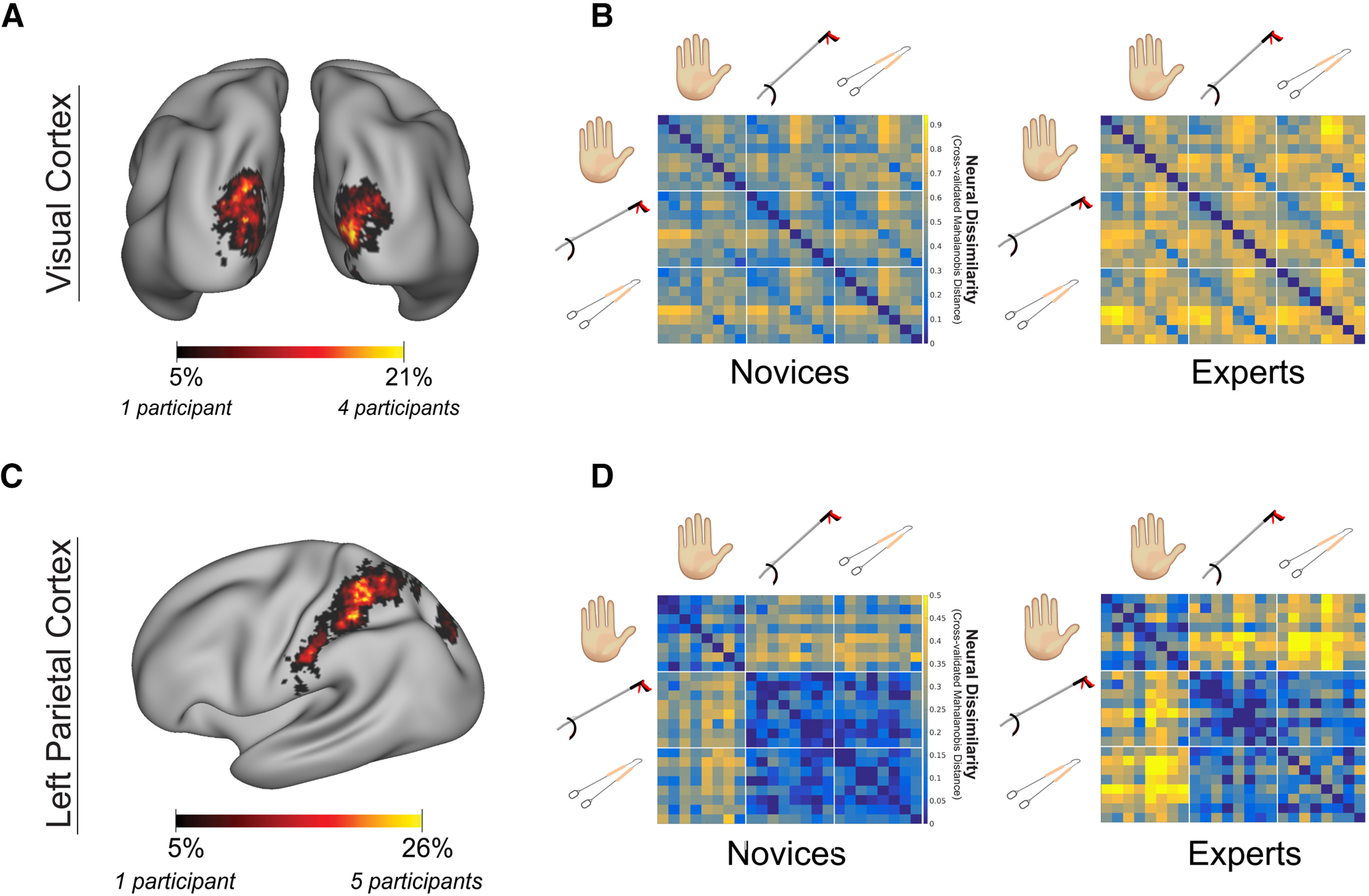
Analyses in visual cortex and left parietal cortex. ***A***, A V1 ROI probability map was constructed for all participants (*n* = 19). Warmer colors represent voxels that were included in a greater number of individual ROIs. ***B***, Group RDMs for V1 showing the pairwise distances (cross-validated Mahalanobis distance) between each video condition. Warmer colors indicate the conditions that evoked more dissimilar patterns of activity. ***C***, A left parietal cortex ROI probability map for all participants (*n* = 19) showing hand and tool selective voxels was defined using independent functional data. ROIs from all participants were superimposed. Warmer colors represent voxels that were included in a greater number of individual ROIs. ***D***, Group RDMs for left parietal cortex showing the pairwise distances (cross-validated Mahalanobis distance) between each video condition. Warmer colors indicate the conditions that evoked more dissimilar patterns of activity.

To understand whether the group differences in effector category distances observed in OTC are driven by differences in the low-level representational structure (e.g., potential differences in eye movements between experts and novices), we repeated the group analysis within a second ROI, V1, as a control (Fig. 3*A*) This analysis revealed no significant group differences in effector category distances within V1 (*F*_(2,17)_ = 0.013, *p* = 0.987; BF_incl_ = 0.330; Fig. 3*B*). However, qualitatively, we observed a trend for a main effect of group (*F*_(1,17)_ = 2.662, *p* = 0.121; BF_incl_ = 0.592) with greater distances in the experts (full statistical report can be accessed at https://osf.io/p4q3y/). Despite not being significant, to highlight that the group differences within OTC are not driven by greater distances in the experts' low-level representational structure captured within V1, we included the average effector category distance in V1 for each participant as a covariate in the OTC analysis. Even when controlling for this low-level representational structure, we still find significant group differences in effector category distances in OTC (significant interaction between effector category distances × group: *F*_(2,17)_ = 11.982, *p* = 0.001; BF_incl_ = 61.216; full statistical report can be accessed at https://osf.io/p4q3y/).

Finally, we considered whether a neural embodiment result (i.e., tools becoming more similar to hands with extensive use) might be observed depending on the laterality of the presented stimuli (left-handed or right-handed), especially considering the experts reported only using the litter picker with their right hand. To test this, the previous RSA approach was repeated in OTC, except this time the video conditions were grouped by their laterality: left-handed or right-handed, i.e., averaged across other video conditions (group laterality RDMs available at https://osf.io/p4q3y/). Nonetheless, we did not observe a significant three-way interaction with the laterality of the stimuli, group and effector category distances (*F*_(2,16)_ = 0.043, *p* = 0.958; BF_incl_ = 0.039), suggesting that the greater distances between hands and tools in the experts is not specific to the way in which the tool is visually experienced in the real world.

### Investigating tool embodiment beyond OTC

While our experiment was specifically designed to leverage the known hand-tool representational relationship of OTC, our video stimuli also activated other regions relating to motor control and planning, providing us the opportunity to perform further exploratory analyses beyond OTC. To test whether a tool embodiment result would potentially be observed within neural structures involved more directly in motor planning for tool use, an exploratory analysis was performed in left parietal cortex (Fig. 3*B*). This analysis revealed significant group differences in effector category distances (interaction between group × effector category distances: *F*_(2,16)_ = 5.058, *p* = 0.020; BF_incl_ = 1.139; Fig. 3*D*), similar to the interaction reported for OTC. However, the group comparisons between each individual effector category distance pair did not reach significance (hand ↔ litter picker: *t*_(18)_ = –0.602, *p* = 0.555, two-tailed; BF_10_ = 0.4; hand ↔ tongs: *t*_(18)_ = 0.440, *p* = 0.116, two-tailed; BF_10_ = 1; litter picker ↔ tongs: *t*_(18)_ = –0.824, *p* = 0.421, two-tailed; BF_10_ = 0.5). Although, on average, experts showed greater distances between hands and litter pickers and hand and tongs compared with novices, similar to what we see within OTC. To verify there is no evidence supporting a neural embodiment result within parietal cortex that contradicts the result within OTC, a one-tailed Bayesian *t* test provided substantial evidence in support of the null hypothesis (BF_10_ = 0.2), i.e., that on average experts do not visually represent an expert tool more similar to hands compared with the novices. Together, while the findings in parietal cortex are weaker than OTC, they are suggestive of a similar pattern and do not provide any evidence supporting tool embodiment.

## Discussion

Here, a fMRI brain decoding technique was used to investigate how similar the representation of a hand is compared with an extensively used tool. This approach allowed us to directly compare hand and tool representations (independent of each other). Contrary to the tool embodiment theory, our findings show that expert tool users do not represent their own tool more similarly to a hand. Instead, experts showed greater dissimilarity distances between visual representations of hands and tools in OTC. Further, using Crawford and Howell's (1998) method, we independently replicated this effect in five of seven individual expert litter pickers, as compared with the novices. Additionally, these group differences were not driven by potential differences in the low-level representation structure, as captured within V1. Despite the experts reporting only using the litter picker with their right hand, we did not find that the group difference in dissimilarity distances between hands and the expert tool was specific to whether the expert tool was viewed as left-handed or right-handed. Additionally, experts showed greater dissimilarity between hands and the non-expert tool (tongs), suggesting that experts have a more distinct representation of general grasping tools. While we did not have clear hypothesis relating to other sensorimotor areas more directly involved in motor planning and control, the exploratory analysis conducted in left parietal cortex provided no evidence supporting a neural embodiment result. Together, our findings in expert tool users provide contradicting evidence to the tool embodiment theory.

There are several potential explanations for the current findings, specifically for how experience with tools leads to a differentiation between hand and tool representations. A primary explanation for the present result is the extensive visual tool experience of the experts. Both short-term (Gauthier et al., 1999; Kourtzi et al., 2005; Op de Beeck et al., 2006; Brants et al., 2016) and long-term (Baker et al., 2007; Chan et al., 2010; McGugin et al., 2012; Dehaene-Lambertz et al., 2018; Gomez et al., 2019) visual experience have been shown to shape representations in visual cortex (for review, see Op de Beeck and Baker, 2010; Harel, 2016). For example, visual training with a category of novel visual objects leads to a differentiation of that category from similar untrained categories (Op de Beeck et al., 2006). Similarly, extensive experience with specific orthographies leads to a distinct representation of those orthographies compared with other orthographies (Baker et al., 2007). This is consistent with our recent work demonstrating that prosthesis usage in amputees leads to greater dissociation of prostheses relative to hands (and tools; Maimon-Mor and Makin, 2020).

Also, while we presume it is the tool representation that has changed in the experts, perhaps it is the representation of the tool action that has changed. Recent work has highlighted the role of OTC in processing observed actions (Tucciarelli et al., 2019). This would explain why experts show greater dissimilarity between hands and both their expert tool (litter picker) and a similar grasping tool on which they did not have prior expertise (tongs). Alternatively, the observed effect for the non-expert tool relative to hands could potentially be driven simply by the shared visual features between the expert and non-expert tools. Indeed, previous research has demonstrated evidence for both of these predictions that OTC encodes information related to stimuli shape (Chen et al., 2018; Wang et al., 2018), as well as the functional/semantic properties of the stimuli (Bracci et al., 2015; Chen et al., 2018).

A second interpretation of the present findings stems from the motor literature which suggests that perhaps the visual hand representation has changed in the experts. Multiple studies have shown that the organizational structure of the sensorimotor hand representation is shaped by the natural statistics of hand usage (Ejaz et al., 2015). Considering the intrinsic functional connectivity between the visual hand area and the sensorimotor hand representation (Tal et al., 2016) and that the expert tool users extensively use their hands to interact with tools, perhaps, the representational shift shown in the experts is driven exclusively by changes in the visual hand representation. This would also explain why the distances relative to both tools changes.

A third interpretation is that the mechanism supporting the increased differentiation of tools from hands observed in experts could be not strictly visual or motor, but rather driven by a larger cognitive mechanism. For instance, in the memory domain, the strengthening of representations is associated with pattern separation, thereby making a new representation less confusable with other memories (Schlichting et al., 2015). Thus, in the present study, for experts to optimally control a hand or tool, the network differentiates these representations, to reduce potential interference and most successfully store and access information.

It is important to note that our experimental design may have several potential limitations. First, while viewing first-person videos during fMRI scans engages visuomotor regions, it did not activate sensorimotor regions (e.g., M1/S1). Considering sensorimotor cortex is more directly involved in the sensory and motor bodily experience, the computations within these neural structures could potentially be different from the pattern observed in OTC and parietal cortex. Unfortunately, the fMRI environment poses unique challenges for active experimental designs involving tool use, and as highlighted above, the actual sensorimotor engagement with the tool provides further confounds that we were eager to avoid. Thus, we cannot rule out the possibility that if subjects were actively involved in tool use during fMRI scans, a different representational structure could have been observed within these regions. Also, it is possible that while watching the videos, experts are mentally simulating actions differently to the novices. In this instance, novice behavior could be more varied in mentally simulating the actions. Previous research is inconclusive on the engagement of OTC during visual and motor imagery (Orlov et al., 2010; Kikuchi et al., 2017). It is also challenging, and perhaps counter-productive, to disentangle cognitive contribution to expert motor learning (Broadbent et al., 2015). Nonetheless, future work is needed to determine whether the motor system produces different representational solutions to those observed here, to support expert tool use, both within and beyond the framework of embodiment.

Finally, it is important to acknowledge the potential limitations of the small sample size used in the present study. Despite our greatest efforts to recruit more litter-picker experts (we originally interviewed 52 candidates for the study), we were only able to secure seven participants. Small sample sizes are known to lead to an overestimation of the actual effect size (Button et al., 2013), and a greater uncertainty around the estimate of the true effect size. Designs with a small sample size are also more susceptible to type II errors. Another problem, related to small sample sizes, is that the distribution of the sample is more likely to deviate from normality, and the limited sample size makes it often impossible to rigorously test the assumption of normality (Ghasemi and Zahediasl, 2012). While we have attempted to account for some of these issues (e.g., by reporting the Bayes factors of the key findings), it is important to place our findings in this limiting context. Where sample size is inherently limited, the advice is to result to replications of the findings (Makin and Orban de Xivry, 2019). As such, here, we used case-study statistics to provide independent replications of our key effect, i.e., greater distances between hands and litter pickers in the experts relative to the novices. Nevertheless, other evidence presented here, and in particular the exploratory analysis in parietal cortex, awaits further confirmation.

In conclusion, while the exact nature for how experience modifies the representational structure is not yet fully understood, the current study offers a striking proof-of-concept for the adult human brain's ability for adaptive plasticity, advancing our current understanding of how categorical selectivity emerges within high level visual cortex. Our findings provide strong evidence that extensive tool use leads to an increased neural differentiation between visual representations of hands and tools. This evidence provides an important alternative framework to the embodiment theory.
